# The chemokine network in lupus nephritis: pathogenesis, targeted therapies, and future directions

**DOI:** 10.3389/fphar.2026.1867426

**Published:** 2026-08-04

**Authors:** Chengye Wang, Liming Chen

**Affiliations:** 1 Queen Mary School, Nanchang University, Nanchang, Jiangxi, China; 2 Department of Rheumatology and Immunology, The First Affiliated Hospital, Jiangxi Medical College, Nanchang University, Nanchang, Jiangxi, China

**Keywords:** chemokine network, future directions, lupus nephritis, pathogenesis, targeted therapies

## Abstract

Lupus nephritis (LN) is a severe complication of systemic lupus erythematosus (SLE), affecting more than 50% of SLE patients and remaining a leading cause of end-stage renal disease (ESRD) worldwide. Current standard therapies—glucocorticoids combined with conventional immunosuppressants—fail to achieve sustained remission in 30%–40% of patients and are associated with substantial toxicities, including infections, osteoporosis, and cardiovascular disease. The chemokine system, composed of small secreted proteins and their G protein-coupled receptors (GPCRs), plays a central role in LN pathogenesis by orchestrating pathogenic immune cell infiltration, renal inflammation, and progressive fibrosis. This review synthesizes preclinical and clinical evidence establishing the chemokine network as a rational therapeutic target in LN. We first outline the clinical burden of LN and the limitations of existing therapies, followed by a systematic overview of chemokine biology (classification, structural interactions, and leukocyte trafficking functions). Next, we dissect the pathogenic roles of chemokines in LN, focusing on their secretion by resident renal cells, regulation of immune cell infiltration, and association with renal fibrosis. Subsequently, we evaluate therapeutic advances targeting chemokines, including small-molecule antagonists, monoclonal antibodies, and upstream pathway interventions, while discussing translational challenges (e.g., preclinical-clinical disconnect, complexity of target selection, safety concerns). Finally, we highlight emerging technologies, opportunities for personalized medicine, and long-term research priorities to facilitate the development of chemokine-based therapeutic strategies for LN.

## Introduction

Lupus nephritis imposes a substantial clinical and economic burden worldwide, with patients under routine clinical management facing high rates of hospitalization, ESRD, and premature mortality (Almaani and Parikh, 2019; Bhusal et al., 2020; Russo and Ryffel, 2024). Epidemiological data indicate that 30%–50% of SLE patients develop LN, and 10%–20% of these progress to ESRD within 10 years of diagnosis (Narayanan et al., 2014; Teoh et al., 2025). Proliferative LN (ISN/RPS class III/IV) represents the most aggressive subtype, with 5-year renal survival rates of 70%–80%, declining to 50% in the presence of crescents (involving >25% of glomeruli) (Cai et al., 2018). A retrospective analysis of 225 LN biopsies revealed renal vascular lesions (RVL) in 44.9% of cases, with severe RVL conferring a 2.3-fold increased risk of composite endpoints (death, ESRD, or ≥30% serum creatinine elevation) (Ma et al., 2025). Membranous LN (class V), characterized by nephrotic syndrome, exhibits 10-year renal survival rates of 60%–70%, yet optimal treatment strategies remain undefined due to limited clinical trial data (Almaani and Parikh, 2019).

Current standard therapy relies on glucocorticoids and conventional immunosuppressants (e.g., mycophenolate mofetil, cyclophosphamide), but significant limitations persist. A scoping review encompassing 15 real-world cohorts demonstrated that 30%–40% of LN patients fail to achieve complete remission with first-line therapy, and 20%–30% experience relapse within 5 years (Teoh et al., 2025). Glucocorticoids increase infection risk by 2- to 3-fold, are associated with a substantially increased risk of osteoporosis-related fracture (Teoh et al., 2025; Almaani and Parikh, 2019). Cyclophosphamide, while effective in proliferative LN, induces ovarian failure in 50% of premenopausal women and elevates bladder cancer risk by 3-fold (OR = 3.1, 95% CI 1.8–5.4) (Almaani and Parikh, 2019). Mycophenolate mofetil offers a superior safety profile but shows diminished efficacy in patients with high chronicity indices (≥4), achieving complete remission in only 25% (Teoh et al., 2025). In pediatric LN, clinical indices such as SLEDAI-R and BILAG-R fail to discriminate ISN/RPS classes, and SDI-R exhibits a sensitivity of only 23.5% for detecting chronic damage (Mina et al., 2016). These constraints underscore the urgent need for novel targeted therapies addressing core pathogenic mechanisms in LN.

Beyond the renal-specific pathology, LN is increasingly recognized as a disease with systemic metabolic and inflammatory derangements. Patients with SLE and LN frequently exhibit intestinal microbiota dysbiosis, metabolic abnormalities (including dyslipidemia and insulin resistance), and an elevated risk of cardiovascular complications (Wang et al., 2026). Emerging evidence suggests that gut microbiota metabolic reprogramming serves as a core driver of chronic inflammation and metabolic disease in the host, and these systemic perturbations may in turn influence the renal inflammatory milieu and disease progression in LN (Wang et al., 2026). While the direct links between gut microbiota-derived metabolites and renal chemokine networks remain to be fully elucidated, this broader systemic perspective underscores the complexity of LN pathogenesis and highlights the potential for integrated therapeutic approaches that address both local renal inflammation and systemic metabolic-immune dysregulation.

Chemokines are 8–12 kDa secreted proteins that orchestrate leukocyte trafficking, activation, and immune homeostasis through interactions with GPCRs (Lira and Furtado, 2012; Miller and Mayo, 2017). Based on the positioning of conserved N-terminal cysteine residues, they are classified into four subfamilies: CC, CXC, CX3C, and C (Miller and Mayo, 2017; Guerreiro et al., 2011). The CC subfamily (e.g., CCL2, CCL3, CCL5) predominantly recruits monocytes, lymphocytes, and eosinophils; the CXC subfamily is divided into ELR^+^ (e.g., CXCL1, CXCL8) and ELR^−^ (e.g., CXCL9, CXCL10, CXCL11) members, promoting neutrophil and T cell migration, respectively (Miller and Mayo, 2017; Balestrieri et al., 2008). The CX3C subfamily (e.g., CX3CL1/fractalkine) mediates monocyte and T cell adhesion and migration, while the C subfamily (e.g., XCL1, XCL2) primarily targets lymphocytes (Lira and Furtado, 2012; Miller and Mayo, 2017).

Chemokine-receptor interactions are characterized by high affinity (Kd = 0.1–10 nM) and ligand-receptor promiscuity, with individual chemokines often binding multiple receptors and vice versa (Bhusal et al., 2020; Miller and Mayo, 2017). For instance, CXCL12 binds both CXCR4 and the atypical chemokine receptor ACKR3/CXCR7, while CCR5 interacts with CCL3, CCL4, and CCL5 (Koenen et al., 2019; Luís et al., 2025). Structural studies reveal that chemokines share a conserved tertiary fold (three-stranded β-sheet/α-helix) but exhibit diverse quaternary structures: CC chemokines frequently form dimers, whereas CXC chemokines may exist as monomers or dimers (Bhusal et al., 2020; Miller and Mayo, 2017). The Duffy antigen receptor for chemokines (DARC), an atypical receptor expressed on erythrocytes and endothelial cells, binds multiple CC and CXC chemokines, functioning as a chemokine sink that regulates plasma levels (Novitzky-Basso and Rot, 2012).

In leukocyte trafficking, chemokines orchestrate a multistep cascade: (1) selectin-mediated tethering and rolling of leukocytes on endothelium; (2) chemokine-induced integrin activation; (3) firm adhesion; and (4) transendothelial migration (Powell et al., 2017; David and Kubes, 2019). During sterile injury, CXCL8a/CXCR2 signaling mediates chemotactic reverse migration of neutrophils, a key step in inflammation resolution (Powell et al., 2017). These fundamental principles of chemokine-mediated leukocyte trafficking are well illustrated in various inflammatory contexts. For example, in infectious disease models, *Campylobacter* jejuni induces dendritic cells to secrete CC (CCL3, CCL4, CCL5) and CXC (CXCL1, CXCL10, CXCL9) chemokines, recruiting monocytes, neutrophils, and T lymphocytes to infection sites (Hu et al., 2012). While these observations derive from infectious disease settings rather than LN, they establish a conceptual framework for understanding how chemokine networks orchestrate leukocyte recruitment—a process that is similarly operational but pathologically dysregulated in the LN kidney. Chemokines also regulate immune cell activation, as demonstrated in studies of monocyte biology: CXCL4, via TNF-α-dependent autocrine signaling, downregulates monocyte CCR1, CCR2, and CCR5 expression, reducing migration toward CC chemokine ligands by 70% in in vitro monocyte studies (Schwartzkopff et al., 2012). While this finding originates from studies of monocyte function in non-LN settings, it illustrates a general principle of chemokine receptor regulation that may have relevance to the altered monocyte trafficking observed in LN. Synergistic interactions between CC and CXC chemokines are common: CXCL8 and CXCL12 enhance monocyte migration toward suboptimal CCL2 concentrations, respectively, through downstream cooperative signaling (ERK1/2 phosphorylation and calcium mobilization) (Go et al., 2008). These findings illuminate the complexity of the chemokine system and its central role in immune homeostasis and inflammation.

Given their central role in orchestrating immune cell recruitment, resident renal cell activation, and fibrotic progression, chemokines and their receptors have emerged as rational therapeutic targets in LN (Li et al., 2026). However, despite a growing body of preclinical evidence and early-phase clinical investigations, no chemokine-targeted therapy has yet been approved for LN, and key translational challenges remain insufficiently addressed in the current literature.

To contextualize the potential role of chemokine-targeted therapies, it is important to consider recent advances in the LN treatment landscape. Beyond conventional immunosuppressants—such as glucocorticoids, mycophenolate mofetil, and cyclophosphamide—several biologic and targeted agents have recently been approved or are in late-stage development for LN (Tanaka et al., 2016). Belimumab, a monoclonal antibody targeting B-cell activating factor (BAFF), has been approved for active LN and has demonstrated improved renal responses in clinical trials (Pohlmeyer et al., 2021). Voclosporin, a calcineurin inhibitor with dual immunosuppressive and podocyte-stabilizing effects, is also approved for LN and offers a steroid-sparing option (Kennedy et al., 2024). Additionally, anifrolumab (an anti-type I interferon receptor antibody) and obinutuzumab (an anti-CD20 B-cell-depleting antibody) have shown promising results in phase II/III trials (Caza et al., 2022; Parodis et al., 2022). Despite these advances, a substantial proportion of patients still fail to achieve complete remission or experience relapse, and none of the currently approved agents directly target chemokine-driven leukocyte recruitment or renal fibrosis (Garal-Pantaler et al., 2024). Chemokine-targeted strategies could therefore complement existing therapies, with potential applications in treatment-refractory disease, relapse prevention, biomarker-guided patient stratification, and anti-fibrotic intervention—though these roles remain to be defined through dedicated clinical investigation.

It is worth noting that several approved LN therapies indirectly modulate chemokine networks. Anifrolumab, by inhibiting type I interferon signaling, downregulates IFN-γ-responsive chemokines including CXCL10 (Caza et al., 2022), while belimumab may affect lymphorganogenic chemokines such as CXCL13 through its impact on B-cell biology (Pohlmeyer et al., 2021). However, these effects are secondary to their primary mechanisms of action rather than direct chemokine targeting. In contrast, true chemokine-targeted strategies—such as CXCR3 or CCR2 antagonists—would directly block the recruitment of pathogenic immune cells to the kidney, potentially offering greater specificity and a distinct mechanism of action that could be combined with existing therapies (Kon et al., 2022). Whether such direct targeting provides additive or synergistic benefit over indirect chemokine modulation remains an open question that requires head-to-head or combination studies.

**FIGURE 1:**
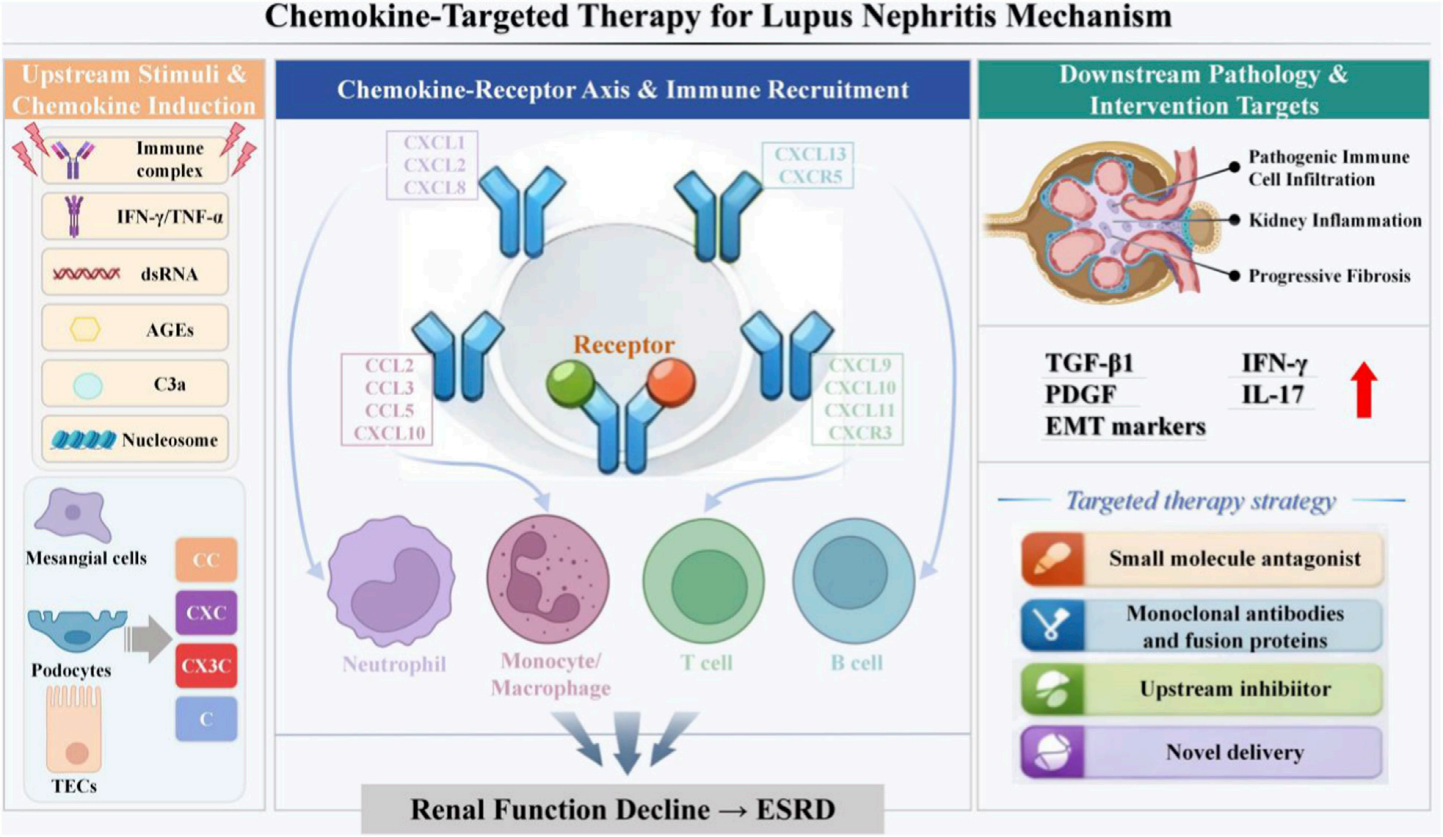
Schematic overview of chemokine-targeted therapeutic mechanisms in lupus nephritis. This diagram delineates the chemokine-receptor axis-mediated pathological cascade of lupus nephritis and corresponding intervention strategies. In the upstream phase, multiple pathogenic stimuli—including immune complexes, proinflammatory cytokines (IFN-γ, TNF-α), double-stranded RNA (dsRNA), advanced glycation end products (AGEs), complement fragment C3a and nucleosomes—activate resident renal cells (mesangial cells, podocytes, tubular epithelial cells [TECs]) to secrete chemokines of distinct subfamilies (CC, CXC, CX3C and C subfamilies). In the central module, secreted chemokines engage their cognate receptors on immune cells, driving directional recruitment and renal infiltration of neutrophils, monocytes/macrophages, T cells and B cells via multiple chemokine-receptor axes. Persistent immune cell infiltration subsequently triggers downstream renal damage, characterized by pathogenic immune cell accumulation, renal inflammation and progressive fibrosis, accompanied by upregulation of profibrotic and proinflammatory mediators (TGF-β1, PDGF, EMT markers, IFN-γ, IL-17), which ultimately leads to renal function decline and progression to end-stage renal disease (ESRD). The right panel summarizes four major categories of chemokine-targeted therapeutic strategies for lupus nephritis: small molecule antagonists, monoclonal antibodies and fusion proteins, upstream inhibitors, and novel delivery systems. Abbreviations: AGEs, advanced glycation end-products; C3a, complement component 3a; CC, chemokine with two adjacent N-terminal cysteines; CXC, chemokine with two N-terminal cysteines separated by one amino acid; dsRNA, double-stranded RNA; EMT, epithelial-mesenchymal transition; ESRD, end-stage renal disease; GPCR, G protein-coupled receptor; IFN-γ, interferon-gamma; LN, lupus nephritis; PDGF, platelet-derived growth factor; SLE, systemic lupus erythematosus; TECs, tubular epithelial cells; TGF-β1, transforming growth factor-beta 1; TNF-α, tumor necrosis factor-alpha.

In this review, we provide an integrative translational framework that bridges chemokine biology, LN pathogenesis, therapeutic targeting, and clinical implementation (Figure 1). Rather than offering a broad narrative summary, we aim to: (i) systematically stratify the available evidence by its direct relevance to LN—distinguishing human LN data, animal model findings, and indirect evidence from other diseases; (ii) critically evaluate the major chemokine axes (CCL2-CCR2, CCL5-CCR5, CXCL9/10/11-CXCR3, CXCL12-CXCR4, and CXCL13-CXCR5) with regard to their pathogenic relevance, therapeutic tractability, and stage of clinical development; and (iii) explicitly address the translational obstacles—including preclinical model limitations, target selection complexity, safety concerns, and biomarker-driven patient stratification—that have hindered clinical success to date. By integrating mechanistic insights with a pragmatic assessment of therapeutic pipelines and regulatory considerations, this review is intended to serve as a resource for renal pharmacologists, rheumatologists, and drug developers interested in the rational advancement of chemokine-targeted strategies for LN. We conclude by proposing a prioritized research agenda and emerging technological opportunities that may facilitate the eventual translation of chemokine network modulation into precision therapies for this challenging disease.

### Pathogenic role of chemokines in lupus nephritis

### Chemokine production by resident renal cells in response to inflammatory stimuli

**FIGURE 2:**
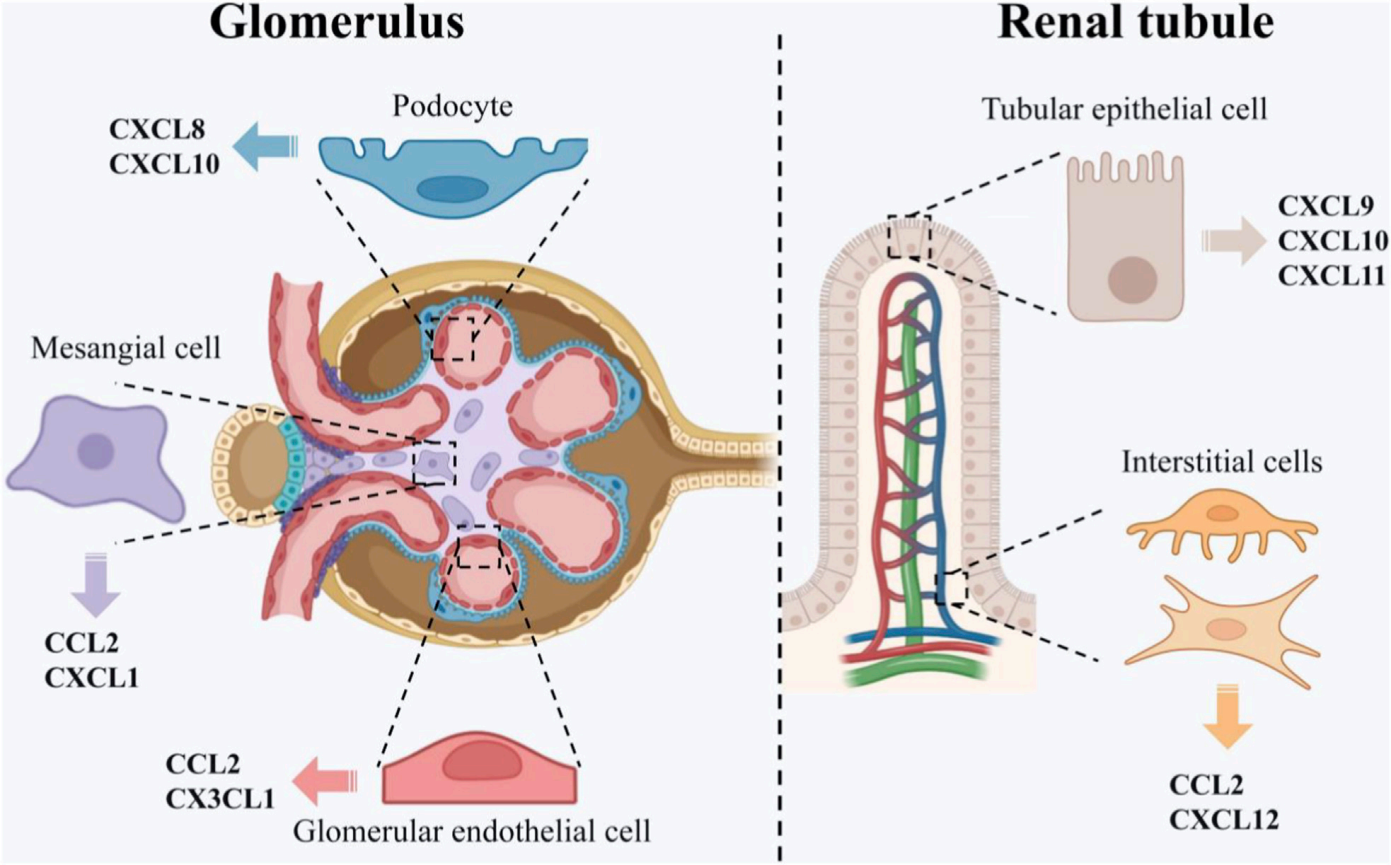
Compartmental chemokine secretion by resident renal cells in glomerular and tubulointerstitial lesions of lupus nephritis. This schematic illustrates the distinct chemokine secretion profiles of intrinsic renal cells across glomerular and tubulointerstitial compartments in the pathogenesis of lupus nephritis. In the glomerular region, three major resident cell subsets participate in local chemokine production: mesangial cells secrete CCL2 and CXCL1; podocytes release CXCL8 and CXCL10; and glomerular endothelial cells produce CCL2 and CX3CL1. In the tubulointerstitial compartment, tubular epithelial cells predominantly generate CXCL9, CXCL10 and CXCL11, whereas renal interstitial cells secrete CCL2 and CXCL12.

Resident renal cells—mesangial cells, podocytes, and tubular epithelial cells (TECs)—are the primary sources of chemokines in LN, responding to inflammatory stimuli such as immune complexes, cytokines, and viral double-stranded RNA (Kanapathippillai et al., 2013; Imaizumi et al., 2012) (Figure 2).

Mesangial cells, central to glomerular inflammation, secrete a broad spectrum of chemokines upon activation. For instance, nucleosomes—major SLE autoantigens—upregulate mRNA expression of CCL2, CCL7, CCL20, CXCL1, CXCL2, and CXCL5 by 5- to 20-fold in primary mesangial cells from (NZBxNZW) F1 mice (Kanapathippillai et al., 2013). *In vivo*, renal CCL2 and CXCL1 levels in B/W lupus mice during nephritic phase are three and four times higher, respectively, than in pre-nephritic phase (Kanapathippillai et al., 2013). Viral double-stranded RNA mimics, via TLR3 and RIG-I signaling, induce MDA5 expression in human mesangial cells, leading to 70- to 100-fold upregulation of CXCL10 mRNA in human mesangial cells following poly (I:C) stimulation (Imaizumi et al., 2012). By comparison, indirect evidence from IgA nephropathy—a disease sharing certain pathogenic features with LN—indicates that galactose-deficient IgA1 stimulates mesangial cells to produce CCL20, recruiting CCR6^+^ Th17 cells to the kidney (Lu et al., 2017). Whether this pathway operates similarly in LN mesangial cells remains to be directly investigated, but it suggests potential mechanistic parallels that warrant future LN-specific studies.

Distinctive features of mesangial cells: Unlike podocytes and TECs, mesangial cells are uniquely positioned within the glomerular tuft and are the primary responders to circulating immune complexes that deposit in the mesangium (Zhang et al., 2023). Their chemokine response is characterized by a broad and robust upregulation of both CC and CXC family members, with particularly strong induction of neutrophil-attracting chemokines (CXCL1, CXCL2) and monocyte-recruiting chemokines (CCL2) (Uyangaa et al., 2015). This broad secretory profile—encompassing chemokines that attract multiple immune cell types simultaneously—distinguishes mesangial cells from the more restricted chemokine repertoires of podocytes and TECs.

Podocytes, specialized cells maintaining the glomerular filtration barrier, also secrete chemokines under inflammatory conditions. Traditionally viewed as passive injury targets, podocytes are now recognized as active participants in inflammation. Indirect evidence from diabetic nephropathy—a disease that shares certain fibrotic pathways with LN but has distinct immune drivers—indicates that advanced glycation end-products (AGEs) stimulate podocytes to produce CCL2, recruiting macrophages to glomeruli (Sasai et al., 2012). Whether similar AGE-driven mechanisms operate in LN podocytes, where immune complexes are the dominant trigger, remains to be directly validated, but this observation highlights the potential of podocyte-derived chemokines as amplifiers of glomerular inflammation. In LN, immune complexes deposited in the subepithelial space activate podocytes via Fcγ receptors, resulting in CXCL8 and CXCL10 secretion (Gao et al., 2020). These chemokines recruit neutrophils and T cells, further compromising the glomerular filtration barrier.

Distinctive features of podocytes: Unlike mesangial cells, which produce a diverse array of chemokines, podocytes exhibit a more restricted chemokine secretory profile, with CXCL8 and CXCL10 being the predominant species induced upon immune complex deposition (Cox et al., 2026; Liu et al., 2023). Furthermore, podocytes are unique among renal cells in that chemokine signaling directly compromises the glomerular filtration barrier by inducing slit diaphragm effacement and podocyte apoptosis—a pathological consequence not observed in mesangial cells or TECs (Patel et al., 2024). This direct link between chemokine signaling and barrier function distinguishes podocytes as critical determinants of proteinuria in LN, a clinical feature that is less directly attributable to the other two cell types.

Tubular epithelial cells, the most abundant renal resident cells, play a pivotal role in tubulointerstitial inflammation in LN. TECs secrete chemokines in response to cytokines, immune complexes, and hypoxia. For example, IFN-γ—a key cytokine in LN—stimulates TECs to produce CXCL10 and CXCL11 via the JAK-STAT pathway (Wang et al., 2021). Co-culture of renal transplant patient-derived CD4^+^ or CD8^+^ T cells with TECs increases CXCL11 and CXCL10 secretion by 5-fold and 3-fold, respectively, an effect abrogated by anti-IFN-γ receptor antibodies (Wang et al., 2021). In renal ischemia-reperfusion injury—an acute injury model that informs general mechanisms of tubular chemokine production—complement component C3a induces CXCL1 and CXCL2 production in TECs via NF-κB signaling (Thurman et al., 2007). While this pathway has not been directly validated in LN, it suggests potential amplification loops that may contribute to chronic tubulointerstitial inflammation in LN and merit future investigation in LN-specific models. TECs also secrete chemokines through exosomes: in models of proteinuric kidney disease, albumin-stimulated TEC-derived exosomes carry CCL2 mRNA, which upon transfer to macrophages enhances their migratory capacity by 4-fold (Lv et al., 2018). Although this mechanism has been primarily studied outside the LN context, it may contribute to tubulointerstitial inflammation in LN if confirmed in LN-specific experimental systems. In diabetic nephropathy, AGEs stimulate TEC-derived CCL2, recruiting macrophages and promoting fibrosis (Sasai et al., 2012). Clinical data support these findings: renal tubular CXCL10 expression in active LN is twice that in remission, and urinary CXCL10 levels correlate significantly with tubulointerstitial activity indices (Gao et al., 2020).

Distinctive features of tubular epithelial cells: TECs are distinguished from glomerular cells by their anatomical location and functional specialization. As the most abundant renal cell type, TECs are the primary source of chemokines driving tubulointerstitial inflammation—a key determinant of chronicity and progressive fibrosis in LN (Ma et al., 2026). Unlike mesangial cells and podocytes, TECs can secrete chemokines through exosome-mediated pathways, enabling paracrine signaling to distal immune cells and fibroblasts (Imaizumi et al., 2012; Casalena et al., 2020). They are also particularly responsive to IFN-γ stimulation via the JAK-STAT pathway, a feature that is less prominent in glomerular cells (Kon et al., 2022). Furthermore, TECs are unique in their capacity to undergo epithelial-mesenchymal transition in response to CXCR4 signaling, directly contributing to the fibrotic process—a feature not observed in mesangial cells or podocytes (Kennedy et al., 2024). This dual role in both chemokine secretion and direct participation in fibrosis positions TECs as central drivers of progressive renal injury in LN.

### Chemokine-mediated recruitment of pathogenic immune cells

In LN, chemokines orchestrate the infiltration of diverse pathogenic immune cells into the kidney, including neutrophils, monocytes/macrophages, T cells, and B cells (Russo and Ryffel, 2024; Gao et al., 2020). Neutrophils are among the earliest infiltrating cells, recruited by ELR^+^ CXC chemokines (CXCL1, CXCL2, CXCL8) secreted by resident renal cells (Gao et al., 2020; Steffen et al., 2018). Neutrophil infiltration in LN patient kidney biopsies is five times higher than in healthy controls, and urinary neutrophil gelatinase-associated lipocalin (NGAL) levels correlate significantly with renal activity (Gao et al., 2020). Neutrophils also cause tissue damage through neutrophil extracellular trap (NET) release, activating macrophages and promoting fibrosis (Jia et al., 2025).

Monocytes/macrophages are key effector cells in renal inflammation and fibrosis in LN, recruited by CC chemokines (CCL2, CCL3, CCL5) and CXC chemokines (CXCL10) (Bambal et al., 2025; Yuan et al., 2015). Macrophage infiltration in active LN kidneys is three times higher than in remission, and macrophage density correlates with chronicity indices (Russo and Ryffel, 2024). Macrophages can polarize into M1 (pro-inflammatory) and M2 (anti-inflammatory/pro-fibrotic) subsets: M1 secretes TNF-α and IFN-γ, exacerbating inflammation; M2 secretes TGF-β1 and PDGF, promoting fibrosis (Anders and Ryu, 2011). In murine LN models, CCR2 antagonists reduce M1 macrophage infiltration and improve renal function (Bambal et al., 2025).

T cells play a central role in LN pathogenesis by secreting pro-inflammatory cytokines and assisting B cells in autoantibody production. Chemokines such as CXCL9, CXCL10, and CXCL11 recruit CXCR3^+^ T cells to the kidney (Russo and Ryffel, 2024; Gao et al., 2020). CXCR3^+^ T cells constitute 40% of renal T cells in LN patients, and their density correlates significantly with renal activity indices (Gao et al., 2020). CD4^+^ T cells are particularly pathogenic: they secrete IFN-γ and IL-17, stimulating resident renal cells to produce additional chemokines (Russo and Ryffel, 2024). Depletion of CD4^+^ T cells in murine LN models attenuates renal inflammation and reduces proteinuria (Russo and Ryffel, 2024). CD8^+^ T cells also contribute to tissue damage through cytotoxicity, infiltrating LN patient kidneys and inducing TEC apoptosis (Gao et al., 2020).

B cells are essential for autoantibody production in LN, with chemokines such as CXCL13 recruiting CXCR5^+^ B cells to the kidney (Russo and Ryffel, 2024; Gao et al., 2020). Urinary CXCL13 levels in LN patients are twice those in controls, and CXCR5^+^ B cells account for 15% of renal B cells (Gao et al., 2020). B cells also secrete chemokines: in murine LN models, B cells produce CCL2 and CCL5, recruiting macrophages and T cells (Russo and Ryffel, 2024). These findings underscore the complex interplay between chemokines and immune cells in LN pathogenesis.

### Role of chemokine signaling in renal inflammation and fibrosis

In LN, chemokine signaling not only regulates immune cell infiltration but also directly promotes renal inflammation and progressive fibrosis (Russo and Ryffel, 2024; Yuan et al., 2015). In glomeruli, chemokine signaling in mesangial cells and podocytes drives cell proliferation, extracellular matrix deposition, and loss of glomerular function. For instance, CCL2 signaling in mesangial cells upregulates TGF-β1 and type I collagen expression by 4-fold and 3-fold, respectively (Kanapathippillai et al., 2013). In podocytes, CXCL10 signaling activates the p38 MAPK pathway, leading to podocyte apoptosis and slit diaphragm effacement (Gao et al., 2020). These changes ultimately culminate in proteinuria, a hallmark clinical feature of LN.

In the tubulointerstitium, chemokine signaling in TECs and fibroblasts promotes inflammation and fibrosis. TEC-secreted chemokines recruit immune cells, which in turn secrete cytokines that induce TECs to undergo EMT. For example, CXCR4 signaling in TECs upregulates α-smooth muscle actin (α-SMA) and downregulates E-cadherin, markers of EMT (Yuan et al., 2015). Fibroblasts are the primary source of extracellular matrix in LN; chemokines such as CCL2 and CXCL12 stimulate fibroblast proliferation and collagen synthesis. In murine LN models, CCR2 antagonists reduce fibroblast activation and collagen deposition (Bambal et al., 2025).

Chemokine signaling also promotes renal fibrosis through cross-talk with other pathways. The CXCL12-CXCR4 axis interacts with the TGF-β1 pathway: CXCR4 signaling upregulates TGF-β1 expression, while TGF-β1 signaling enhances CXCR4 expression (Yuan et al., 2015). This positive feedback loop exacerbates fibrosis. The CCR5 axis cross-talks with the NF-κB pathway: CCR5 signaling activates NF-κB, which in turn upregulates pro-inflammatory cytokines and chemokines (Bambal et al., 2025). Clinical data support these pathways: renal CXCR4 and TGF-β1 expression in LN patients with advanced fibrosis is three times higher than in those with early-stage disease (Yuan et al., 2015).

Beyond direct effects on renal cells, chemokine signaling in immune cells also contributes to fibrosis. M2 macrophages secrete TGF-β1 and PDGF, activating fibroblasts (Anders and Ryu, 2011). T cells secrete IFN-γ and IL-17, increasing chemokine production by resident renal cells and further recruiting immune cells (Russo and Ryffel, 2024). B cells produce autoantibodies that form immune complexes, activating complement and stimulating chemokine secretion (Gao et al., 2020). These findings highlight the complexity of the chemokine signaling network in LN pathogenesis, necessitating therapeutic strategies that simultaneously target immune cell infiltration and resident renal cell activation.

The five chemokine-receptor axes summarized in Table 1 differ not only in their cellular sources and target immune populations but also in the strength of evidence linking each axis to human LN pathogenesis and their relative tractability for therapeutic intervention. The CXCL9/10/11-CXCR3 axis is distinguished by consistent elevation of CXCL10 in active LN patient sera and urine, with strong correlation with histologic activity (Tanaka et al., 2016). The CCL2-CCR2 axis is supported by robust upregulation in LN kidneys and preclinical efficacy of receptor blockade (Pohlmeyer et al., 2021; Gao et al., 2020). The CCL5-CCR5 and CXCL12-CXCR4 axes are mechanistically compelling, particularly for their roles in fibrosis and chronicity, though much of the evidence derives from non-LN models (Go et al., 2008; Kennedy et al., 2024). The CXCL13-CXCR5 axis, while conceptually attractive given the central role of B cells in LN, has the weakest direct evidence base (Tanaka et al., 2016). This differential evidence landscape has direct implications for therapeutic development: axes with strong human biomarker validation and preclinical efficacy are prioritized for near-term clinical translation, whereas those with limited LN-specific data require further mechanistic investigation before clinical advancement. The following section evaluates the therapeutic agents targeting each of these axes, their stage of development, and the challenges that have hindered clinical progress.

**TABLE 1:** Major chemokine-receptor axes implicated in lupus nephritis pathogenesis.

Chemokine axis	Major source cells in LN kidney	Target immune cells	Pathological role	Evidence type*
CCL2-CCR2	Mesangial cells, TECs, podocytes	Monocytes, macrophages	Recruitment of monocytes/macrophages; promotion of glomerular and tubulointerstitial inflammation; fibroblast activation	Human LN biopsies (↑ expression), LN murine models (↑ expression, antagonist efficacy), indirect evidence from other renal diseases
CCL5-CCR5	TECs, infiltrating T cells	T cells, macrophages	Recruitment of T cells and macrophages; promotion of interstitial inflammation and fibrosis	Human LN biopsies (↑ expression), LN murine models (antagonist efficacy), genetic association studies
CXCL9/10/11-CXCR3	TECs, mesangial cells, infiltrating immune cells	Th1 cells, CD8^+^ T cells, NK cells	Recruitment of CXCR3^+^ T cells; promotion of glomerular and tubulointerstitial inflammation	Human LN biopsies and urine (↑ levels, correlation with activity), LN murine models (antagonist efficacy)
CXCL12-CXCR4	TECs, fibroblasts, infiltrating immune cells	TECs (autocrine), immune cells	Promotion of EMT and fibrosis in TECs; retention of immune cells in tissues	Human LN biopsies (↑ expression in fibrotic regions), murine fibrosis models, limited LN-specific validation
CXCL13-CXCR5	Infiltrating immune cells, possibly TECs	B cells, T follicular helper cells	Recruitment of B cells to kidney; promotion of autoantibody production and immune complex formation	Human LN urine (↑ levels), limited LN tissue data; largely indirect evidence

* Evidence type key: Human LN, biopsies = direct evidence from patient kidney tissue; LN, murine models = evidence from MRL/lpr or NZB × NZW F1 mouse studies; Indirect evidence = findings from other diseases or non-LN, experimental systems that suggest mechanistic parallels.

### Therapeutic strategies targeting chemokine pathways: research progress

Current therapeutic strategies targeting the chemokine network in LN can be most effectively categorized by the specific chemokine-receptor axis they engage. For each axis, we summarize its biological role in LN, the evidence level supporting its pathogenic relevance, representative therapeutic agents, stage of clinical development, and key limitations. Table 2 provides a comprehensive overview of major chemokine axes and their corresponding therapeutic strategies.

**TABLE 2:** Chemokine-targeted therapeutic strategies for lupus nephritis: representative agents and development status.

Target axis	Representative agent	Mechanism	Disease/model tested	Development stage	Key limitations
CCL2-CCR2	Cenicriviroc	Dual CCR2/CCR5 antagonist (small molecule)	NASH (phase IIb); murine renal fibrosis models	Phase IIb completed (NASH); no LN trials	Infection risk due to monocyte mobilization; pathway redundancy may limit single-axis efficacy
CCL5-CCR5	Maraviroc	CCR5 antagonist (small molecule)	HIV (approved); murine LN models	Approved for HIV; preclinical in LN	Opportunistic infection risk; potential effects on regulatory T cell function
CXCL9/10/11-CXCR3	AMG487 (CXCR3 antagonist); anti-CXCL10 mAb	Small-molecule receptor antagonist; monoclonal antibody	Murine LN models; psoriasis (anti-CXCL10, phase II)	Preclinical in LN; phase II in psoriasis	CXCR3 expression on tregs may affect immune regulation; ligand redundancy limits single-ligand targeting
CXCL12-CXCR4	AMD3100 (plerixafor); LY2510924	Small-molecule antagonist (AMD3100); peptide antagonist (LY2510924)	Stem cell mobilization (approved); murine fibrosis models	Approved for stem cell mobilization (AMD3100); preclinical in fibrosis	Hematopoietic effects; leukocytosis; limited long-term safety data in chronic use
CXCL13-CXCR5	CXCR5 antagonists; anti-CXCL13 antibodies (in development)	Small-molecule or biologic agents	Preclinical only	Preclinical	Broad effects on humoral immunity; infection risk; no LN-specific data
Upstream pathways (NF-κB, MAPK, JAK-STAT)	PDTC (NF-κB); SB203580 (p38 MAPK); tofacitinib (JAK)	Pathway inhibitors	Ischemia-reperfusion injury; RA (tofacitinib, approved); murine LN models	Preclinical in LN; approved in other indications (tofacitinib)	Broad immunosuppression; off-target effects; safety concerns with chronic use

### CCL2-CCR2 axis

Biological role in LN: CCL2 (MCP-1) is secreted by mesangial cells, TECs, and infiltrating immune cells in response to immune complexes and pro-inflammatory cytokines. It recruits CCR2^+^ monocytes and macrophages to the kidney, promoting glomerular and tubulointerstitial inflammation (Pohlmeyer et al., 2021). In LN patient biopsies, CCL2 expression correlates with macrophage infiltration and disease activity (Tanaka et al., 2016).

Evidence level: CCL2 upregulation has been consistently demonstrated in LN patient kidney biopsies and murine LN models (Pohlmeyer et al., 2021). Urinary CCL2 levels correlate with renal disease activity and predict treatment response in some cohorts (Tanaka et al., 2016).

Therapeutic agents: Cenicriviroc—a dual CCR2/CCR5 antagonist—has been evaluated in NASH, showing reduction in macrophage infiltration and fibrosis (Go et al., 2008). Preclinical studies in murine models of renal fibrosis have also shown that CCR2 antagonism reduces macrophage accumulation and attenuates inflammatory injury (Gao et al., 2020). Other CCR2-selective antagonists are in preclinical development.

Stage of development: Phase IIb completed in NASH; no LN-specific clinical trials reported to date (Ratziu et al., 2020).

Limitations: Given the role of CCR2 in monocyte egress from bone marrow and tissue surveillance, systemic antagonism may increase susceptibility to infections. Additionally, redundancy in chemokine pathways may limit the efficacy of single-axis targeting.

### CCL5-CCR5 axis

Biological role in LN: CCL5 (RANTES), produced by TECs and infiltrating T cells, recruits CCR5^+^ T cells and macrophages to the kidney. CCR5^+^ macrophages are enriched in LN kidneys and correlate with chronicity indices (Go et al., 2008). Genetic association studies have linked CCR5 polymorphisms to LN susceptibility and progression.

Evidence level: CCR5 expression is elevated in LN kidney biopsies, and CCR5^+^ macrophages are prominent in active disease (Go et al., 2008). Preclinical studies in murine LN models support the pathogenic role of this axis.

Therapeutic agents: Maraviroc, a small-molecule CCR5 antagonist approved for HIV-1 infection, has been studied in murine LN models, with reported attenuation of renal inflammation and fibrosis (Go et al., 2008). Anti-CCR5 monoclonal antibodies have also been explored in preclinical settings.

Stage of development: Approved for HIV; preclinical studies in LN models; no LN clinical trials registered to date.

Limitations: CCR5 blockade may impair T cell trafficking to sites of infection, potentially increasing susceptibility to opportunistic pathogens. Moreover, CCR5 is also expressed on regulatory T cells, and its inhibition could theoretically affect immune homeostasis.

### CXCL9/10/11-CXCR3 axis

Biological role in LN: CXCL9, CXCL10, and CXCL11 are IFN-γ-inducible chemokines that recruit CXCR3^+^ Th1 cells, CD8^+^ T cells, and NK cells to inflamed tissues. In LN, CXCL10 is consistently elevated in serum and urine during active disease, and CXCR3^+^ T cells are prominent in kidney infiltrates (Tanaka et al., 2016). Urinary CXCL10 correlates with renal activity indices, supporting its utility as a biomarker and therapeutic target (Erez et al., 2024).

Evidence level: Strong evidence from LN patient cohorts demonstrates elevated CXCL10 in active disease and its correlation with histologic activity (Tanaka et al., 2016). Preclinical studies in murine LN models confirm that CXCR3 antagonism reduces T cell infiltration and attenuates proteinuria (Russo and Ryffel, 2024).

Therapeutic agents: Small-molecule CXCR3 antagonists (e.g., AMG487) have shown efficacy in reducing T cell infiltration and proteinuria in murine LN models (Russo and Ryffel, 2024; Tanaka et al., 2016). Anti-CXCL10 monoclonal antibodies have demonstrated activity in preclinical LN models and have been evaluated in other autoimmune conditions such as psoriasis, where they reduced disease activity (Tanaka et al., 2016).

Stage of development: Preclinical in LN; phase II completed in psoriasis (anti-CXCL10); no LN-specific trials reported.

Limitations: CXCR3 is also expressed on regulatory T cells, and its blockade may affect immune regulatory functions (Moreno Ayala et al., 2023). Additionally, CXCL9, CXCL10, and CXCL11 bind the same receptor, so targeting a single ligand may be insufficient to fully block the axis.

### CXCL12-CXCR4 axis

Biological role in LN: CXCL12 (SDF-1) and its receptor CXCR4 are constitutively expressed in the kidney and are upregulated under inflammatory and fibrotic conditions. In TECs, CXCR4 signaling promotes EMT and TGF-β1 production, contributing to interstitial fibrosis (Kennedy et al., 2024). CXCR4 is also expressed on immune cells and mediates their retention in tissues.

Evidence level: CXCR4 upregulation has been demonstrated in fibrotic regions of LN kidneys and in murine models of renal fibrosis (Kennedy et al., 2024). However, most mechanistic evidence derives from non-LN models of chronic kidney disease, and LN-specific data remain limited.

Therapeutic agents: AMD3100 (plerixafor), a small-molecule CXCR4 antagonist, is approved for hematopoietic stem cell mobilization. LY2510924, a peptide-based CXCR4 antagonist, has been evaluated in preclinical models of fibrosis (Kennedy et al., 2024). Both agents block CXCL12-CXCR4 signaling.

Stage of development: Approved for stem cell mobilization (AMD3100); preclinical in fibrosis models (LY2510924); no LN-specific clinical trials reported (Huang et al., 2025; Cho et al., 2015).

Limitations: CXCR4 is critical for hematopoiesis and immune cell homing. Systemic inhibition may cause leukocytosis and potentially affect immune surveillance. Long-term safety data in chronic use settings are limited.

### CXCL13-CXCR5 axis

Biological role in LN: CXCL13, a B-cell chemoattractant, recruits CXCR5^+^ B cells to the kidney and is elevated in the urine of LN patients (Tanaka et al., 2016). Given the central role of B cells in autoantibody production and immune complex formation, this axis represents a rational therapeutic target in LN.

Evidence level: Urinary CXCL13 levels are elevated in LN patients and correlate with renal B cell infiltration (Tanaka et al., 2016). However, direct evidence from therapeutic targeting of this axis in LN is currently lacking.

Therapeutic agents: Several CXCR5 antagonists and anti-CXCL13 antibodies are in early-stage preclinical development, though none have advanced to LN-specific clinical evaluation (Yang et al., 2025; Pan et al., 2022).

Stage of development: Preclinical.

Limitations: CXCR5 is also expressed on a subset of T follicular helper cells, which are important for germinal center reactions. Targeting this axis may affect humoral immunity more broadly, potentially increasing infection risk.

### Upstream pathway interventions: a cross-axis strategy

In addition to direct targeting of individual chemokines or receptors, modulating upstream signaling pathways that regulate chemokine production offers a complementary approach. Pathways such as NF-κB, MAPK, and JAK-STAT converge on chemokine gene transcription and can be pharmacologically inhibited to reduce the production of multiple chemokines simultaneously (Kon et al., 2022; Kanapathippillai et al., 2013).

NF-κB inhibitors: In models of renal ischemia-reperfusion injury, the NF-κB inhibitor pyrrolidine dithiocarbamate (PDTC) reduces TEC chemokine production (Kanapathippillai et al., 2013). Extending these mechanistic insights to LN, subsequent preclinical studies in murine LN models have demonstrated that PDTC attenuates renal inflammation and fibrosis (Kanapathippillai et al., 2013). However, this latter finding derives from a single preclinical study, and the therapeutic potential of NF-κB inhibition in LN—particularly given its broad immunosuppressive effects—requires further validation in LN-specific models and careful safety assessment.

MAPK inhibitors: In cultured mesangial cells stimulated with nucleosomes—a model system relevant to LN—the p38 MAPK inhibitor SB203580 reduces chemokine production (Pohlmeyer et al., 2021). However, direct evidence of therapeutic efficacy in LN is more limited; to our knowledge, only a single preclinical study has reported that SB203580 attenuates renal inflammation and proteinuria in murine LN models (Pohlmeyer et al., 2021). These encouraging but preliminary observations warrant replication in independent LN models.

JAK-STAT inhibitors: IFN-γ signaling via JAK-STAT pathways drives chemokine production in renal TECs. In TEC culture systems stimulated with IFN-γ, the JAK inhibitor tofacitinib reduces chemokine production (Kon et al., 2022). Beyond these *in vitro* observations, tofacitinib has demonstrated clinical efficacy in rheumatoid arthritis, though this indirect evidence should not be directly extrapolated to LN (Kon et al., 2022). Preclinical studies in murine LN models have reported that tofacitinib attenuates renal inflammation and fibrosis (Kon et al., 2022). These findings collectively support further investigation of JAK inhibition in LN, but the extent to which *in vitro* and non-LN *in vivo* data predict human LN efficacy remains uncertain.

Beyond pharmacological inhibition of these canonical signaling cascades, emerging evidence indicates that post-translational modifications—particularly SUMOylation (small ubiquitin-like modifier conjugation)—represent an additional layer of regulation for the NF-κB, MAPK, and JAK-STAT pathways that drive chemokine synthesis and release in inflammatory conditions (Y. Wu, Z. Chen, Y. Hu, G. Zengin, Q. Zhang, M.-Y. Li, W. Sun, Interdiscip. Med. 2026, 4, e70120. https://doi.org/10.1002/inmd.70120). SUMOylation modulates the transcriptional activity, nuclear translocation, and protein stability of key signaling molecules within these pathways, thereby influencing the magnitude and duration of chemokine production. Although the direct role of SUMOylation in LN has not been extensively characterized, this regulatory mechanism may offer novel therapeutic opportunities for modulating chemokine networks at the post-translational level and warrants investigation in LN-specific contexts.

Advantages and limitations: Upstream pathway interventions can block the production of multiple chemokines simultaneously, potentially achieving broader efficacy than single-axis targeting. However, they carry greater risks of off-target effects and immunosuppression, as these pathways regulate diverse biological processes beyond chemokine production (Kanapathippillai et al., 2013). The development of pathway-selective inhibitors and combination with direct chemokine targeting may help balance efficacy and safety.

### Emerging technologies

Emerging therapeutic modalities—including gene therapy, extracellular vesicles (EVs), and nanotechnology—are being explored to enhance the delivery and efficacy of chemokine-targeted interventions.

Gene therapy: RNA interference strategies, such as siRNA targeting CXCR3 or CXCR4, have shown efficacy in reducing chemokine receptor expression and attenuating inflammation in preclinical models (Russo and Ryffel, 2024). However, efficient and kidney-specific delivery systems remain a major hurdle for clinical translation.

Extracellular vesicles: EVs derived from mesenchymal stromal cells or engineered to carry chemokine inhibitors have been investigated as delivery vehicles in various disease models (Lv et al., 2018). Preclinical studies suggest that EV-based delivery may enhance target cell specificity and reduce systemic exposure, though these approaches remain at an early experimental stage for LN applications (Lv et al., 2018).

Nanotechnology: Nanoparticle-based delivery systems have been explored to improve renal targeting of chemokine receptor antagonists (Gao et al., 2020). While this approach may reduce systemic toxicity and enhance local drug accumulation, most evidence to date derives from non-LN models, and safety and efficacy in LN require direct investigation.

Despite their theoretical advantages, these emerging technologies face substantial translational barriers, including manufacturing complexity, immunogenicity concerns, uncertain biodistribution, and limited LN-specific preclinical validation (Gao et al., 2020; Lv et al., 2018). Further development and rigorous evaluation in LN-relevant models are warranted before clinical application can be considered.

### Comparative assessment of chemokine axes for therapeutic development

Based on the available evidence, the relative therapeutic potential of the five major chemokine axes discussed in this review differs substantially, and several factors should inform target prioritization for future clinical development.

The CXCL9/10/11-CXCR3 axis currently benefits from the strongest LN-specific evidence. CXCL10 is consistently elevated in serum and urine of active LN patients, correlates with histologic activity indices, and urinary levels predict treatment response in some cohorts (Tanaka et al., 2016). Preclinical efficacy of CXCR3 antagonists in murine LN models has been demonstrated, and anti-CXCL10 antibodies have shown clinical activity in other autoimmune conditions such as psoriasis (Tanaka et al., 2016). This axis therefore represents the most clinically advanced and best-validated target for near-term LN trials. However, ligand redundancy (CXCL9, CXCL10, and CXCL11 all signal through CXCR3) suggests that receptor blockade may be more effective than single-ligand neutralization (Green et al., 2025).

The CCL2-CCR2 axis also has robust preclinical support, with consistent upregulation in LN kidneys and therapeutic efficacy of CCR2 antagonists in murine models (Go et al., 2008; Pohlmeyer et al., 2021). Clinical proof-of-concept in other inflammatory diseases (e.g., NASH) further supports the tractability of this target (Go et al., 2008). However, LN-specific clinical data are lacking, and CCR2’s role in monocyte mobilization from bone marrow raises safety concerns that may limit dosing in chronic settings (El et al., 2022).

The CCL5-CCR5 axis is mechanistically well-characterized in LN, with CCR5^+^ macrophages enriched in diseased kidneys and genetic associations linking CCR5 polymorphisms to disease progression (Go et al., 2008). The availability of maraviroc, an approved CCR5 antagonist with a well-established safety profile in HIV, facilitates repurposing (Wang et al., 2022). However, broader safety considerations—particularly the risk of opportunistic infections and CCR5 expression on regulatory T cells—may temper enthusiasm, and clinical trial data in LN are entirely lacking (Zhang et al., 2026).

The CXCL12-CXCR4 axis is compelling for its dual role in inflammation and fibrosis, making it an attractive target for patients with progressive chronicity (Kennedy et al., 2024; Meng et al., 2025). However, the evidence base is largely derived from non-LN models of chronic kidney disease, and the hematopoietic effects of CXCR4 inhibition (including leukocytosis and stem cell mobilization) pose substantial safety barriers for chronic use in LN patients. The therapeutic window for this target may therefore be narrower than for other axes.

The CXCL13-CXCR5 axis is conceptually attractive given the central role of B cells in LN pathogenesis and autoantibody production (Tanaka et al., 2016). However, direct therapeutic evidence is almost entirely lacking, with no clinical-stage agents specifically evaluated in LN. Given the availability of approved B-cell-targeted therapies (e.g., belimumab, obinutuzumab), it remains uncertain whether direct CXCR5 targeting offers any distinct advantage over existing B-cell-directed approaches (Pu et al., 2024; Desai et al., 2026).

In summary, the CXCR3 and CCR2 axes currently appear to be the most advanced and tractable targets for near-term clinical development in LN, supported by consistent biomarker data and preclinical efficacy. The CCR5 and CXCR4 axes face greater safety barriers but remain mechanistically attractive for specific patient subsets (e.g., those with predominant fibrosis or chronicity). The CXCR5 axis, while biologically plausible, requires more foundational evidence before clinical advancement can be justified. Given the functional redundancy inherent in the chemokine system, combination strategies targeting multiple axes may ultimately be required to achieve optimal efficacy, particularly in patients with refractory or relapsing disease.

## Clinical translation: challenges and opportunities

### The preclinical-clinical translational gap: limitations of animal models in predicting human efficacy

The translational gap between preclinical findings and clinical efficacy is a major challenge in the development of chemokine-targeted therapies for LN. LN animal models such as MRL/lpr and NZBxNZW F1 mice recapitulate certain features of human LN, including immune complex deposition, renal inflammation, and fibrosis. However, several limitations reduce their predictive value for human efficacy. For instance, MRL/lpr mice develop severe autoimmunity and nephropathy by 6 months of age, but their pathogenesis is driven by Fas deficiency, which is not a primary factor in human LN. Similarly, NZBxNZW F1 mice develop nephropathy by 12 months of age, with milder disease severity than human LN.

Beyond these general limitations, the two most commonly used LN mouse models differ substantially from each other and from human LN in several key aspects that critically affect their utility for translational research.

Autoantibody profiles: MRL/lpr mice produce predominantly anti-dsDNA and anti-Sm autoantibodies, with a strong Th1-dominant immune response (Zheng et al., 2023). NZB × NZW F1 mice exhibit a more diverse autoantibody repertoire, including anti-dsDNA, anti-histone, and anti-C1q antibodies, which more closely resembles the autoantibody spectrum observed in human proliferative LN (Casalena et al., 2020). However, neither model fully recapitulates the breadth of autoantibody specificities seen in human SLE, and the temporal evolution of autoantibody development differs substantially between murine models and patients (Tomalla et al., 2023).

Renal pathology: MRL/lpr mice develop severe diffuse proliferative glomerulonephritis (ISN/RPS class IV) with prominent crescent formation and rapid disease progression, whereas NZB × NZW F1 mice develop a mixed proliferative and membranous phenotype (class III/IV with superimposed membranous features) that progresses more slowly (Casalena et al., 2020). The tubulointerstitial inflammation and fibrosis—which are critical determinants of long-term renal outcomes in human LN—are more pronounced in MRL/lpr mice but still occur with a different temporal progression and topographic distribution than in humans (Zhang et al., 2025). Importantly, the human ISN/RPS classification system, which guides clinical treatment decisions, cannot be directly applied to murine models due to fundamental differences in glomerular architecture and immune cell distribution.

Fibrotic progression: Both models develop interstitial fibrosis over time, but the molecular pathways driving fibrosis—including TGF-β1, PDGF, and CTGF signaling—are less robustly activated in murine models than in human LN (Casalena et al., 2020). Furthermore, the kinetics of fibrotic progression differ: in humans, fibrosis accumulates over years of chronic inflammation, whereas in murine models it develops over weeks to months (Bousamaki et al., 2025). This compressed timeline may not accurately reflect the chronic, relapsing nature of human LN and limits the utility of these models for studying anti-fibrotic interventions.

Local immune microenvironment: The composition of immune infiltrates differs significantly between murine models and human LN. For instance, NK cell infiltration is more prominent in MRL/lpr mice, whereas plasma cell infiltration and tertiary lymphoid structure formation are more characteristic of human LN (Casalena et al., 2020). Additionally, the stromal cell response to injury—including fibroblast activation, pericyte differentiation, and extracellular matrix remodeling—exhibits species-specific differences in chemokine receptor expression and signaling kinetics that may affect the translation of chemokine-targeted therapies. The cytokine milieu also differs: while IFN-γ and TNF-α are elevated in both murine models and human LN, the relative contributions of Th1, Th2, and Th17 responses vary substantially between species and models (Claßen et al., 2025).

These fundamental differences in disease drivers, autoantibody repertoire, histopathological features, fibrotic pathways, and immune microenvironment should be carefully considered when interpreting preclinical data and designing translational studies. Efforts to develop more representative preclinical models—including humanized mice expressing human chemokines and chemokine receptors, genetically modified mice with inducible disease, and patient-derived xenograft models—are ongoing and may improve the predictive value of preclinical LN research (Casalena et al., 2020). However, until such models are validated, findings from conventional murine models should be interpreted with appropriate caution, and therapeutic strategies showing efficacy in these models should be prioritized for clinical evaluation based on a combination of mechanistic plausibility, biomarker evidence from human LN, and safety considerations.

Another limitation of animal models is species-specific differences in chemokine signaling. For example, human CXCL10 is a potent T cell chemoattractant, whereas its murine ortholog exhibits lower potency. Additionally, CCR5 expression patterns differ between species: in human, CCR5 is expressed on T cells and macrophages, whereas in mice its expression is more broadly distributed across immune cell subsets (Patino-Martinez et al., 2026). These interspecies differences may lead to false-positive or false-negative results in preclinical studies when murine findings are extrapolated to human LN. It is important to emphasize that such comparative observations derive from cross-species immunological studies rather than LN-specific investigations; however, they highlight the need for caution in interpreting preclinical data and underscore the value of human tissue-based validation.

Beyond model-specific limitations, several additional factors contribute to the frequent failure of promising preclinical chemokine-targeted interventions in clinical translation. First, the chemokine system exhibits substantial functional redundancy, such that inhibiting a single chemokine or receptor may be insufficient to block pathogenic leukocyte recruitment when alternative axes can compensate (Cecchinato et al., 2023). Second, pharmacokinetic challenges—including rapid clearance, poor tissue penetration, and inadequate target engagement in the kidney—are often underestimated in preclinical models that do not fully recapitulate human drug distribution (Pilati and Howard, 2020). Third, the timing of intervention in animal studies (typically initiated before or shortly after disease onset) rarely mirrors the clinical setting, where patients present with established disease. Fourth, safety concerns—particularly infection risk and off-target immunosuppression—have led to caution in dose selection and trial design, potentially limiting efficacy. These translational barriers underscore the need for more predictive preclinical models, improved pharmacokinetic/pharmacodynamic modeling, and biomarker-driven patient selection in future trial design.

Three-dimensional *in vitro* models, including kidney organoids and microfluidic devices, are emerging as complementary tools to animal models for studying chemokine signaling in a human-relevant context (Doeser et al., 2026). Although much of the technological development in this area has originated from cancer research and other non-renal fields, these platforms are now being adapted for kidney disease applications. For example, human induced pluripotent stem cell (iPSC)-derived kidney organoids have been shown to secrete chemokines in response to immune complexes—a response that can be modulated by chemokine-targeted interventions (Gaykema et al., 2024). Similarly, microfluidic devices designed to model the kidney microenvironment enable real-time observation of leukocyte migration and have provided proof-of-concept data supporting the role of CXCL10 as a potent human T cell chemoattractant (Ren et al., 2023). However, these technologies remain in early stages of development for LN research, and their predictive value for human therapeutic responses has not yet been systematically validated.

Despite their promise, three-dimensional *in vitro* models have limitations. Organoids lack renal vasculature and immune components, and microfluidic devices are complex to fabricate and operate. Furthermore, production costs are high, and their predictive value for human efficacy remains unclear (Baptista et al., 2022). Continued refinement of these models and systematic validation against human LN data will be essential to establish their predictive utility.

### Complexity of target selection

The complexity of the chemokine system poses a major challenge for therapeutic target selection in LN (Bhusal et al., 2020; Luís et al., 2025). The chemokine system exhibits functional redundancy, with multiple chemokines and receptors mediating the same biological effect. For instance, CXCL9, CXCL10, and CXCL11 all bind CXCR3 and recruit T cells to the kidney (Gao et al., 2020). This redundancy implies that targeting a single chemokine or receptor may be ineffective, as other molecules can compensate.

Another challenge in target selection is the context-dependent dual role of chemokines. Depending on the environment, chemokines can exert both pro-inflammatory and anti-inflammatory effects. For example, CXCL12 promotes EMT and fibrosis in TECs but facilitates renal repair after acute kidney injury (Yuan et al., 2015). Furthermore, chemokines exert distinct effects on different cell types: CXCL10 recruits T cells to the kidney but also promotes B cell activation (Gao et al., 2020). These context-dependent effects complicate target selection, necessitating a balance between benefit and risk.

To address these challenges, researchers employ systems biology approaches to map the LN chemokine network (Bhusal et al., 2020; Luís et al., 2025). These approaches, including transcriptomics, proteomics, and metabolomics, identify key chemokine pathways in LN. For instance, transcriptomic analysis of LN patient kidney biopsies identified CXCL9-CXCR3 and CCL2-CCR2 pathways as critical drivers of renal inflammation (Gao et al., 2020; Yuan et al., 2015). Similarly, urinary proteomic analysis identified CXCL10 and CCL2 as biomarkers of disease activity (Gao et al., 2020). These systems biology approaches facilitate the selection of targets that are critical to LN pathogenesis with minimal off-target effects.

Another strategy to address target complexity is combination therapy. Combining agents targeting multiple chemokine pathways can overcome redundancy and enhance efficacy. For example, combining a CXCR3 antagonist with a CCR2 antagonist reduces renal inflammation and fibrosis in murine LN models, compared with single agents (Russo and Ryffel, 2024). Furthermore, combining chemokine-targeted therapies with conventional immunosuppressants may enhance efficacy while reducing toxicity. For instance, combining a CXCR3 antagonist with mycophenolate mofetil reduces renal inflammation in murine LN models, compared with mycophenolate mofetil alone (Russo and Ryffel, 2024).

### Balancing efficacy and safety: risks associated with systemic inhibition of leukocyte trafficking

Balancing efficacy and safety is a major challenge in the development of chemokine-targeted therapies for LN (Russo and Ryffel, 2024; Dong et al., 2017). Systemic inhibition of leukocyte trafficking may increase susceptibility to infections, as leukocytes are critical for host defense. For example, CCR5 antagonists reduce circulating CD4^+^ T cell counts, increasing the risk of opportunistic infections. Similarly, CXCR4 antagonists mobilize hematopoietic stem cells, potentially leading to neutropenia and increased susceptibility to bacterial infections (Berg et al., 2016).

Another safety concern is off-target effects of chemokine-targeted therapies. For instance, CXCR4 antagonists can cross the blood-brain barrier and affect neuronal function, leading to neurocognitive side effects (Berg et al., 2016). Additionally, CCR2 antagonists affect monocyte migration to the bone marrow, potentially causing anemia (Bambal et al., 2025). These off-target effects may limit the clinical application of chemokine-targeted therapies.

To address these safety concerns, researchers are developing tissue-specific chemokine-targeted therapies. For example, kidney-targeted nanoparticles deliver CXCR3 antagonists, reducing systemic exposure and off-target effects (Sun et al., 2019). Additionally, prodrugs activated only in the kidney can deliver chemokine-targeted therapies to the site of pathology (Sun et al., 2019). These tissue-specific strategies may enhance efficacy while reducing safety risks.

Another strategy to balance efficacy and safety is the use of biomarkers to monitor therapeutic response and toxicity. For instance, serum CXCL10 levels can monitor CXCR3 antagonist efficacy, and absolute lymphocyte counts can monitor infection risk (Gao et al., 2020). Additionally, biomarkers such as urinary NGAL can monitor renal toxicity (Gao et al., 2020). These biomarkers enable clinicians to adjust dosing, balancing efficacy and safety.

### Biomarkers and patient stratification: identifying subgroups most likely to respond to specific chemokine-targeted therapies

Biomarkers and patient stratification are critical for the successful translation of chemokine-targeted therapies in LN (Gao et al., 2020; Li JR. et al., 2025). LN is a heterogeneous disease, with variable patient responses to therapy. Identifying patient subgroups most likely to respond to specific chemokine-targeted therapies can enhance efficacy and reduce costs.

Serum and urinary chemokines are potential biomarkers for LN. For instance, serum CXCL10 levels in patients with active LN were three times higher than in remission, and urinary CXCL10 levels correlate with renal activity indices (Gao et al., 2020). Furthermore, serum CCL2 levels in LN patients with advanced fibrosis are twice those in early-stage patients (Gao et al., 2020). Recent clinical evidence further supports the utility of circulating chemokine profiling in SLE, with significant correlations reported between serum chemokine levels and clinical manifestations of disease activity (Dziedzic et al., 2026). These chemokines can be used to stratify patients and identify those most likely to respond to chemokine-targeted therapies.

Immune cell subsets represent another class of LN biomarkers. For example, CXCR3^+^ T cells constitute 40% of renal T cells in active LN, and their density correlates significantly with renal activity indices (Gao et al., 2020). Additionally, CCR5^+^ macrophages constitute 30% of renal macrophages in LN patients with advanced fibrosis, and their density correlates with chronicity indices (Bambal et al., 2025). These immune cell subsets can be used to stratify patients and identify those most likely to respond to CXCR3 or CCR5 antagonists.

Genetic biomarkers are also under investigation in LN. For instance, single nucleotide polymorphisms (SNPs) in the CXCR3 gene are associated with increased LN susceptibility (OR = 1.5, 95% CI 1.2–1.8) (Gao et al., 2020). Additionally, SNPs in the CCR5 gene are associated with increased risk of renal fibrosis (Bambal et al., 2025). These genetic biomarkers can be used to stratify patients and identify those most likely to respond to chemokine-targeted therapies.

Patient stratification based on these biomarkers can enhance the efficacy of chemokine-targeted therapies. For example, LN patients with high serum CXCL10 levels exhibit a threefold higher response rate to CXCR3 antagonists than those with low levels (Gao et al., 2020). Similarly, LN patients with high renal CCR5^+^ macrophage density exhibit a twofold higher response rate to CCR5 antagonists than those with low density (Bambal et al., 2025). These findings underscore the importance of biomarkers and patient stratification in the translation of chemokine-targeted therapies for LN.

## Future directions in chemokine research for lupus nephritis

### Emerging technologies in chemokine research

Emerging technologies are revolutionizing chemokine research in LN, with several approaches in preclinical development (Mardpour et al., 2019; Kiaie et al., 2019). These include single-cell RNA sequencing (scRNA-seq), spatial transcriptomics, and organoid models, enabling the study of chemokine signaling in a human-relevant context.

Single-cell RNA sequencing is a powerful tool for studying the heterogeneity of chemokine signaling in LN. It enables the identification of cell-specific chemokine expression patterns and signaling pathways (Mou et al., 2024; Li Y. et al., 2025). For example, scRNA-seq of LN patient kidney biopsies has identified subpopulations of mesangial cells, podocytes, and TECs expressing distinct chemokine profiles (Mardpour et al., 2019). Additionally, scRNA-seq has identified immune cell subsets expressing distinct chemokine receptors (Mardpour et al., 2019). These findings facilitate the selection of cell-specific targets for chemokine-targeted therapies.

Spatial transcriptomics is another emerging technology in chemokine research. It enables the study of the spatial distribution of chemokine expression in kidney biopsies (Li Y. et al., 2025). For instance, spatial transcriptomics of LN patient kidney biopsies has identified specific regions of chemokine expression in glomeruli and tubulointerstitium (Mardpour et al., 2019). Furthermore, spatial transcriptomics has revealed co-localization of chemokines and their receptors in specific renal regions (Mardpour et al., 2019). These findings enhance our understanding of the local regulation of chemokine signaling in LN.

Organoid models are also being used to study chemokine signaling in LN. Human iPSC-derived kidney organoids recapitulate the complex structure and cellular interactions of the kidney, enabling the study of chemokine signaling in a human-relevant environment. For example, kidney organoids secrete chemokines in response to immune complexes, a response modulated by chemokine-targeted therapies (Gessaroli et al., 2026). Additionally, organoid models enable the study of chemokine-targeted therapy effects on renal function. These findings facilitate the selection of therapeutic regimens most likely to be effective in humans.

### Potential for personalized medicine

Personalized medicine represents a potential future direction for chemokine-targeted therapies in LN (Al Riyami et al., 2025; Segura Tudela et al., 2025). LN is a heterogeneous disease, with variable patient responses to therapy. Personalized medicine utilizes biomarkers to select therapies for individual patients, enhancing efficacy and reducing costs.

One personalized medicine strategy is the use of genetic biomarkers to select chemokine-targeted therapies. For instance, LN patients carrying CXCR3 gene SNPs exhibit a threefold higher response rate to CXCR3 antagonists than those without SNPs (Gao et al., 2020). Similarly, LN patients carrying CCR5 gene SNPs exhibit a twofold higher response rate to CCR5 antagonists than those without SNPs (Bambal et al., 2025). These genetic biomarkers can be used to select therapies for individual patients.

Another personalized medicine strategy is the use of immune cell subsets to select chemokine-targeted therapies. For example, LN patients with high renal CXCR3^+^ T cell density exhibit a threefold higher response rate to CXCR3 antagonists than those with low density (Gao et al., 2020). Similarly, LN patients with high renal CCR5^+^ macrophage density exhibit a twofold higher response rate to CCR5 antagonists than those with low density (Bambal et al., 2025). These immune cell subsets can be used to select therapies for individual patients.

Liquid biopsy is another potential personalized medicine strategy for LN. Liquid biopsies, including blood and urine samples, enable real-time monitoring of chemokine levels and immune cell subsets. For instance, serial monitoring of serum CXCL10 levels can assess CXCR3 antagonist efficacy and guide dose adjustments as needed (Gao et al., 2020). Additionally, serial monitoring of urinary CCL2 levels can assess renal fibrosis progression and guide dose adjustments (Gao et al., 2020). These liquid biopsies enable clinicians to tailor therapy for individual patients.

### Long-term perspectives and research priorities

The long-term prospects for chemokine-targeted therapies in LN remain to be determined, but continued preclinical and clinical investigation is warranted. However, several research priorities must be addressed to achieve clinical translation.

The first research priority is the development of more predictive LN animal models. Current animal models have several limitations that reduce their predictive value for human efficacy. The development of animal models that recapitulate human LN pathogenesis—including immune complex deposition, renal inflammation, and fibrosis—is critical for the translation of chemokine-targeted therapies. Furthermore, the development of humanized animal models expressing human chemokines and receptors may enhance the predictive value of preclinical studies.

The second research priority is the identification of novel chemokine targets in LN. Current chemokine-targeted therapies focus on a limited set of targets (e.g., CXCR3, CCR2, CCR5). The identification of novel chemokine targets, such as CXCR4 and CXCL12, may expand the therapeutic landscape for LN (Gao et al., 2020). Additionally, the identification of novel chemokine signaling pathways, such as CXCL9-CXCR3, may facilitate the development of more effective therapies (Gao et al., 2020).

The third research priority is the development of combination therapies for LN. Combination therapies targeting multiple chemokine pathways can overcome redundancy and enhance efficacy. The development of regimens combining chemokine-targeted therapies with conventional immunosuppressants may enhance efficacy while reducing toxicity (Russo and Ryffel, 2024). Furthermore, the development of combination regimens incorporating chemokine-targeted therapies is warranted.
